# The Meaning of Life and Death in the Eyes of Frankl: Archetypal and Terror Management Perspectives

**DOI:** 10.5964/ejop.4689

**Published:** 2021-08-31

**Authors:** Claude-Hélène Mayer, Nataliya Krasovska, Paul J. P. Fouché

**Affiliations:** 1 Department of Industrial Psychology and People Management, University of Johannesburg, Johannesburg, South Africa; 2 Faculty of Cultural Studies, European University Viadrina Frankfurt (Oder), Frankfurt, Germany; 3 European University Viadrina Frankfurt (Oder), Frankfurt, Germany; 4 Department of Psychology, University of the Free State, Bloemfontein, South Africa; Nelson Mandela University, Port Elizabeth, South Africa

**Keywords:** archetypes, Carl Gustav Jung, Ernest Becker, meaning, psychobiography, terror management, Viktor Frankl

## Abstract

This article aims to uncover the meaning of life and death across the lifespan of the extraordinary person, Viktor E. Frankl (1905–1997). Frankl was purposively sampled due to his international acclaim as an Austrian neurologist and psychiatrist, who later became famous as a holocaust survivor and the founder of logotherapy. Through his approach of “healing through meaning,” he became the founder of the meaning-centred school of psychotherapy and published many books on existential and humanistic psychology. The study describes the meaning of life and death through two theoretical approaches: the archetypal analysis based on C.G. Jung’s and C.S. Pearson’s work and a terror management approach based on the melancholic existentialist work of Ernest Becker. The methodology of psychobiography is used to conduct the psycho-historical analysis of the interplay of archetypes and death annihilation anxiety throughout Frankl’s lifespan. The article evaluates how archetypes and death anxiety interacts and how they built meaning in different stages of Frankl’s lifespan. The theories are discussed and illustrated in the light of Viktor E. Frankl’s life.

*When we are no longer able to change a situation,*

*we are challenged to change ourselves.*

— *Viktor E. Frankl*

*Without suffering and death, human life cannot be complete.*

— *Viktor E. Frankl (1946/2006, p. 67)*

Viktor E. Frankl was born in 1905 in Vienna, Austria. In his childhood he already appeared to be thinking deeply about the nature of life. Aged four, for example, he already expressed his anxiety of the transitory nature of life (Frankl, 2000). This experience of deep philosophical questions at an early stage continued and led, during his teenage years to a deeper interest in in natural philosophy, such as Wilhelm Ostwald and Gustav Theodor Fechner (Frankl, 2000). Frankl gave his first lecture on the meaning of life in the philosophical study group of a community college at the age of 15 (Längle, 2013) which he anchored in different psycho-philosophical thoughts, such as of Freud’s psychoanalysis and Adler’s Individual Psychology.

During his life, Frankl published scientific articles and founded the magazine “Man in Everyday Life—A Magazine for the Distribution and Application of Individual Psychology” (Längle, 2013). In 1927, however, Adler excluded Frankl from the Association for Individual Psychology (Frankl, 2000), since their basic ideas regarding humans motives and drives different strongly (Wong, 2017). Aged 25, he completed his studies and worked as a psychiatrist and psychotherapist at the Psychiatric University Clinic (Frankl, 2000), and later on at the Rothschild Hospital in Vienna. In 1941, Frank married nurse Tilly Grosser (Frankl, 2000). Soon after, he and his entire family were deported to Theresienstadt (Frankl, 2000). All of his family members died in concentration camps (Frankl, 2000). After the liberation, he worked as a primary physician in the Vienna Polyclinic (Längle, 2013). In 1946 he published the book “A Psychologist Experiences the Concentration Camp,” later entitled “Man’s Search for Meaning” (Frankl, 2000). Soon he started to lecture internationally. In 1947, he married the nurse Eleonore Schwindt. He dedicated his life to psychology, the exploration of meaning in life and sharing his experiences of a concentration camp survivor. Frankl died in Vienna in 1997 (Längle, 2013).

This article aims to uncover the meaning of life and death within the life of Viktor E. Frankl (1905–1997). More specifically, the study describes the meaning of life and death in Frankl’s life through two theoretical stances: the archetypal analysis based on Jung’s (1969, 1971, 1980) and Pearson’s (2012) and Pearson and Marr’s (2002, 2007) work, as well as the terror management theory (TMT) based on Becker (1967, 1973).

Firstly, this article presents four archetypes—the Seeker, Destroyer, Caregiver, and Magician—and their expression in the context of finding meaning in life’s adversities and during suffering. Buffardi (2019) highlights that archetypes are innate and pre-existent to the psychic development of the individual. He criticised that archetypes are viewed from an existential psychology perspective rather than created by the individual interacting with the environment. Buffardi (2019) explains how the interpretation of archetypes can support existential therapy and the client’s internal maps. Based on this approach, the authors argue that archetypal analysis within an existential positive psychology approach can be helpful in analysing the meaning of life and death—as two most important anchors in TMT of Becker (1967, 1973) in extraordinary individuals. Secondly, the articles explain the TMT and relate it to the life and death experiences of Frankl, interpreting his life, thoughts, experiences and actions in the context of selected TMT aspects. Frankl’s life was further analysed by using Alexander’s indicators as means to extract salient data (see Schultz, 2005). These indicators are referred to in the article, where relevant. Overall, the article illustrates and evaluates how archetypes, death anxiety, and terror management interact or interplay with one another and how they built meaning in different stages of Frankl’s lifespan.

## The Archetypal Theory of Jung and Pearson

Jung is the pioneer of archetypal theory and has defined archetypes as images, symbols, and themes of the collective consciousness (Jung, 1969, 1971, 1980). Thus, archetypes can be both positive (represented in the light aspect) and negative (represented in the shadow aspect; Jung, 1971; Snider, 2013). Archetypes are universal patterns with individual and culture-specific nuances (Buffardi, 2019; Hall, 1989). According to Jung (1971), archetypal events, such as birth, death, separation of parents, initiation, marriage or the union of opposites, usually bring about archetypal patterns (Jung, 1980). Jung (1971) is primarily associated with 12 archetypes, namely the sage, the innocent, the explorer, the ruler, the creator, the caregiver, the magician, the hero, the rebel, the lover, the trickster, and the orphan.

Various studies have focused on different patterns as archetypal formations (Bolen, 2014; Moore & Gillette, 1991; Myss, 2013). Also, psychobiographies have used archetypal theories to analyse the life of extraordinary individuals (Mayer, 2020). In this study, the archetypes of Pearson (2012) and Pearson and Marr (2002, 2007) were used for several reasons: Firstly, Pearson describes archetype and their development in-depth, up to their fullest potential (Pearson, 2012), thereby providing a well-established framework of analysis in psychobiography. Secondly, four out of the 12 archetypes of Pearson (2012), namely the Seeker, the Destroyer, the Caregiver, and the Magician were chosen, as they allow a deeper insight in the process of meaning-seeking and meaning-making in different life contexts.

### The Seeker: Search for a Better Life

The goal of the Seeker archetype is to explore the new inner and the outer world, based on yearning: “Cinderella longs for her prince to come; Geppetto longs to have a child. Telemachus searches for Odysseus; the prince searches for a great treasure” (Pearson, 2012, p. 133). According to Jung (see Dunne, 2015), the Seeker often feels floating, setting a course between light and dark but ultimately moves along by unseen currents deep within. The Seeker tends to search for their identity and struggles to fulfil their true potential (Pearson & Marr, 2012). The call to the journey can be an urge to function at a higher or deeper level, to find a more significant way to live, to find out who the person is beyond the social norms. The quest often starts with the need to make choices as the hero feels confined or empty (Pearson, 2012). The yearning is fulfilled when giving birth to the true self and realising that “the real issue is expanding our consciousness beyond the boundaries of Ego reality” (Pearson, 2012, p. 134). To find the inner truth, one must “answer the call to embark on the heroic life” (Pearson, 2012, p. 134).

### The Destroyer: Metamorphosis

Pearson (2012) proposes the archetype of the Destroyer as a representation of the unwanted change rather than an active choice. It is particularly relevant for the life of Viktor E. Frankl as he was challenged to deal with alienation, suffering, and human mortality, which he could not control or change. The Destroyer is an independent archetype (Pearson, 2012), associated with tragedy or loss to rebuild one’s own life (Pearson & Marr, 2007). The goal of the Destroyer is growth and metamorphosis through a life situation caused by the death of a close person or a sudden awareness of human mortality (Pearson, 2012). Entering the journey requires an encounter with fear and recognition that the reality is not neat and in human control (Pearson, 2012). It is essential to trust the process of letting go, whether chosen or not, as part of the metamorphosis for which the Destroyer aims (Pearson & Marr, 2007). The key to the journey is to sacrifice something in order to be healed or to save the world (Pearson, 2012). This sacrifice has many reasons: freedom from attachment, openness to transformation, compassion in ourselves and others (Pearson, 2012).

### The Caregiver: Love, Compassion and Sacrifice

The archetype of the Caregiver represents people who gain satisfaction from caring for others and who value kindness (Pearson & Marr, 2007). Essential in the Caregiver’s story is an element of power or hierarchical distinction, which is used to care for another in need (Rutledge & Atlee, 2013). It is the Caregiver’s story that pushes us to tend to those in need, to take care of those less able, and to nurture. It is typical for the Caregiver to create community by helping people feel that they are valued and by encouraging nurturing relationships (Pearson, 2012), and it is reflected in the Tree of Life, which continually feeds and sustains. Caregivers know who they are and what they aspire. The caring attitude towards others is more potent than the predisposition for self-preservation. Examples of this archetype are Christ and Gandhi, who are asked to die for others, a cause or faith (Pearson, 2012).

### The Magician: Transformation and Healing

The power of the Magician archetype is to transform reality by changing their attitude, thoughts, and reactions (Pearson, 2012). People claiming the Magician role in society have been known as shamans, witches, healers, fortune-tellers, priests or priestesses, and contemporarily as doctors or psychologists. The Magician understands that the structure of consciousness governs the outer circumstances; therefore, the Magician uses prayer, meditation, psychotherapy or other methods to ensure that their state of mind is clear and corresponds to their life purpose (Pearson & Marr, 2002). When they establish order in their inner world, it becomes simple for them to have order in the external world, working on synchronicity (Jung, 1971, 1980). Synchronistic events draw us to experiences that match our inner realities (Pearson, 2012) and support the experience of reciprocation of internal and external worlds, realising complex connections.

## Ernest Becker’s existentialist TMT

TMT is an existentialist theory, referring to “a philosophy that confronts the human situation in its totality to ask what the basic condition of human existence is and how man can establish his own meaning out of these conditions” (Barrett, 1958, p. 126). The TMT, developed by the social-anthropologist Becker, based on the research of Otto Rank (1950), assumes that human behaviour is motivated by conscious and unconscious mortality salience. Questions such as ”Why am I here?,” “Who am I?,” “How do I relate to other people and to nature itself?,” but also “How much control do I have over my life?” and “What happens when life ends?” are core questions of human’s existence. TMT argues that individuals deal with these questions by either reasoning consciously or using unconscious mechanisms that help them cope with life and death (Pyszczynski, Greenberg, Koole, & Solomon, 2010, p. 2). TMT’s early developments are rooted in the works of Yalom (1980), Greenberg, Koole, and Pyszczynski (2004), and Koole, Greenberg, and Pyszczynski (2006). Recent developments in existential social psychology have focused particularly on the impact of death and mortality on mental health and social functioning (Greenberg et al., 2004; Vail & Routledge, 2020), as well as on meaning in life (Steger, 2012).

### Directions in TMT Theory

There are three main strings in TMT theory which have been discussed over the past decades and which are introduced in the following section:

Firstly, according to Becker’s existentialist theory, nearly every human being fears death, and this death anxiety drives individuals to adopt worldviews that protect their worthiness, their self-esteem, and their sustainability. This idea has been supported by other researchers (e.g., Van Kessel, Den Heyer, & Schimel, 2020) and is described as *mortality salience hypothesis* (Rosenblatt, Greenberg, Solomon, Pyszczynski, & Lyon, 1989). Based on this, individuals believe that they play a meaningful role in the world and create a meaningful life, as a means to overcoming their existential anxiety to die. Examples include writing a book, living up to one’s standard and worldview, and believing in an afterlife (Vail et al., 2012). Becker (1967) highlights that the TMT supports individuals in dealing with uncertainty regarding their own worldview and building amicable ways in relationships with others, which might be interpreted as a contribution to a peaceful society.

Becker, as an existential psychoanalytical anthropologist, further argues that humans’ primary death anxiety drives them to distraction, to the illusion of immortality and the denial of death. Further, he assumes that cultural worldviews aim at providing the potential for humans to have value and giving them hope of transcending death (Pyszczynski et al., 2010). By denying death, individuals cope with existential terror, manage social cohesion and individual approaches to resolve the terror. Fisher (2020) adds that even 45 years after the work of Kübler-Ross (1975) and her criticism that humans do not deal enough with death, death is denied in many societies. Furthermore, culture, as well as religion, support the idea of overcoming death through transcendence by providing ideas of overcoming it in either a symbolic (continuing as part of something greater and longer-lasting than oneself) or literal way (believing that life continues after death; Pyszczynski et al., 2010).

Secondly, another hypothesis in TMT is the *anxiety-buffer hypothesis,* which assumes that certain psychological constructs, such as high self-esteem, secure relational attachment, or religious faith, can buffer mortality salience (Vail et al., 2012). Juhl (2019) emphasises that investing in cultural belief systems, maintaining close relationships and self-esteem all impact on minimising negative affect and anxiety.

Thirdly, Hayes, Schimel, Arndt, and Faucher (2010) have described the *death thought accessibility hypothesis* in TMT, which highlights that if an element buffers against death awareness (e.g., stereotyping, prejudiced thought), death-related cognitions will increase unconsciously. For example, individuals who experience threat as their worldview are more prone to death thoughts, showing that a strong and solid faith in own values and cultural assumptions buffers the awareness of mortality, while a weak faith and belief in one’s own values and cultural assumptions increases death awareness (Schimel, Hayes, Williams, & Jahrig, 2007).

### The Shadow and the Light Side of TMT

Van Kessel, Den Heyer, and Schimel (2020) assert that TMT supports individuals to reflect about life and death, while Vail et al. (2012) state that the awareness of mortality can increase motivations in positive ways, such as enhance their physical health, or prioritise growth-oriented goals, adhere to positive standards and beliefs, develop themselves in peaceful, charitable communities, foster open-mindedness and growth-oriented behaviours, as well as positive and supportive relationships. According to the authors, the encounter with death can be a potentially enriching and impactful experience and increase the promotion of well-being globally—it can become a positive health resource. In this context, the meaning of life can, while increasing death awareness, change from extrinsically motivated goals towards more intrinsically motivated goals (Vail et al., 2012). If ascribed meaning is changed or disrupted, it might create anxiety. While meaning in life is a strong coping mechanism with regard to tragedy or trauma, it can also be seen as a symbolic mechanism to deal constructively with death and mortality awareness (Heine, Proulx, & Vohs, 2006).

## Design and Method

A psychobiography can be defined as a biography written on a significant individual, based on the use of psychological theory or models (Du Plessis, 2017; Fouché & Van Niekerk, 2010; Mayer & Maree, 2017; Ponterotto, 2017a, 2017b). It is a subdivision of psychohistory, with the researcher implementing psychological theory and socio-historical methods in order to uncover and reconstruct the life of the subject under study (Ponterotto, 2017a, 2017b).

This psychobiography utilised a “multiple-lens” approach. The two main approaches included: 1) archetypal analysis, based on Jung’s (1969, 1971, 1980), Pearson’s (2012) and Pearson and Marr’s (2002, 2007) theories on archetypes and 2) TMT, based on the existentialist work of Becker (1973), to uncover the meaning of life and death across Frankl’s lifespan. Through the exploration of archetypes and death anxiety, the researchers illustrate and evaluate the interrelationship of meaning-making through these two theoretical lenses at different points in Frankl’s life. The methodology thereby entailed longitudinal life-history research (Fouché, 2015; Fouché & Van Niekerk, 2005, 2010) with a qualitative and single-case research design (Yin, 2018).

### Victor Frankl: The Psychobiographical Subject

One of the initial steps in conducting a psychobiography is to select the psychobiographical subject (Du Plessis, 2017; Mayer, 2017). Frankl was chosen as the subject for this study utilising a non-probability, purposive sampling technique. Purposive sampling relies on the researcher's judgement in determining the desired characteristic attributes to be investigated and ensuring the richness of data (Strydom & Delport, 2005). Frankl was chosen due to his contemporary, historical and theoretical significance (Längle, 2013). Another important factor to consider is the amount of readily and publically available primary and secondary sources based on the subject’s life. Although the quantity of available sources is considered important, the quality of the data is equally important in terms of the subject’s life-narrative (Mayer, 2017; Mayer & Kőváry, 2019). Another guideline to be followed includes the ethical considerations surrounding a psychobiographical study on a renowned subject (Du Plessis, 2017; Mayer & Kőváry, 2019; Ponterotto & Reynolds, 2019).

### Collection of Data Sources

Psychobiographical researchers use two main sources of data to extract their evidence, namely primary and secondary sources (Du Plessis, 2017; Fouché & Van Niekerk, 2005; Saccaggi, 2015). The authors included primary sources, such as personal documents, diary entries, letters, recorded items, and autoethnographical books (Yin, 2018), as well as secondary sources, such as hearsay reports, articles and biographies related to the subject (Fouché & Van Niekerk, 2005; Yin, 2018). Only publically available sources were utilised, which reduces ethical risk to the study since no private data are being exposed which have been been exposed to the public before (Ponterotto & Reynolds, 2019).

### Extraction and Analysis of Data

A psychobiography can be considered well-suited and sophisticated if the researchers can extract relevant evidence on the subject and apply it to the psychological theories utilised in the study, and vice versa. Generalising the case findings or evidence to the theories used to interpret the case refers to analytical generalisation (Yin, 2018).

Evidence in the data was extracted, categorised, and analysed using two theories and a strategy to identify salience (i.e., the identification of significant evidence). Alexander’s (1988) nine identifiers or indicators of saliencewere the first strategy that assisted the researchers in making sense of the vast amount of sources and data gathered, especially evidence derived from personal interviews with the subject, and first-person or primary written documents. Although Alexander (1988) originally developed this method, Demorest (2005) further refined the strategy. These nine indicators of salience include frequency, primacy, emphasis, isolation, uniqueness, incompletion, and error or distortion (see Alexander, 1988), which assisted in revealing the subject’s personal experience (Demorest, 2005) and formulating tentatively proposed hypotheses (Elms, 2007). The researchers collected data on Frankl with the following question in mind: *Which sections of the data collected will allow for an in-depth understanding, evaluation, and illustration of meaning-making in the interaction between the archetypes and existential death anxiety of Frankl?* The researchers conceptualised Frankl’s meaning-making of life and death by making use of Jung and Pearson, as well as Becker’s theoretical constructs.

### Quality Criteria

To enhance the study’s trustworthiness and its consistency or reliability (Creswell, 2013; Silverman, 2017; Yin, 2018), the following strategies were operationalised:

1. Data triangulation and analyst triangulation (Yin, 2018) and the use of two psychological approaches (i.e., theoretical triangulation).
2. Prolonged, extensive engagement with the biographical and historical data on the subject.
3. The researchers upheld a constant awareness of the cautious alliance (Ponterotto & Moncayo, 2018) between them, as psychobiographers, and the biographical relationship with Frankl.
4. Frankl’s unique socio-historical, cultural, and political contexts were investigated to facilitate a contextually accurate interpretation of his life.
5. The researchers strove for procedural rigour through the systematic application of established strategies for extracting salient data via the salience indicators of Alexander (1988).
6. The researchers considered the close connection between methodological competence and ethical considerations in psychobiography, as suggested by Ponterotto and Reynolds (2019).

## Findings

In the following section, the authors apply the theories utilised in this study to Frankl’s life.

### Archetypal Theory in Frankl’s life: The Seeker

The call of the Seeker occurs in Frankl’s life during childhood and interpenetrates his life path until he finds responses to his questions about the meaning of life and death. As a child, Frankl was called “The Thinker” as he asked adults many questions concerning life and death (Frankl, 2015). The feeling of emptiness brought him to the journey of exploring if “the transitory nature of life might destroy its meaning” (Frankl, 2000, p. 29). In his autobiography, he discusses his habit of daily self-contemplation (Frankl, 2000) to not only explore the meaning of life for humankind, but in particular the meaning of his own life for humankind (Frankl, 2000).

Frankl’s journey to an understanding of the meaning of life shows ambition, perfectionism, and suppressed desire to be appreciated. In his book “Autobiography” (Frankl, 2000), Alexander’s (1988) pointer of Isolation can be found when he suddenly begins speaking about himself: “I have always been amused when I heard that others had an idea that I had had a long time before” (Frankl, 2000, p. 33). In the preface of his book “Man’s Search for Meaning,” Frankl mentions the immense global popularity of this book which expands its original context while speaking to human’s need for search for meaning (Krasovska & Mayer, 2021).

Through the popularity of his writings, Frankl experienced appreciation, driving him to explore meaning of his own life:

In some respects, it is death itself that makes life meaningful. Most importantly, the transitoriness of life cannot destroy its meaning because nothing from the past is irretrievably lost. Whatever we have done, or created, whatever we have learned and experienced—all of this we have delivered into the past. There is no one and nothing that can undo it (Frankl, 2000, p. 29).

Therefore, the understanding of death is transformed through the Seeker's journey in the life of Frankl. Death is not the enemy; it becomes part of an eternal life.

### Archetypal Theory in Frankl’s life: The Destroyer

The Destroyer archetype in Frankl’s life is expressed when he becomes a “renegade of the spirit” (Frankl, 2000, p. 62) and by emphasising his opinion in the Society for Individual Psychology about how individual psychology had still to free itself from psychologism (Frankl, 2000). However, the Destoyer archetype was also active in his strong experience of the loss of his family which led to 10 years of void and disorientation and the stopping of the logotherapy development (Längle, 2013). His experience of powerlessness, however, was followed by new accomplishments, turning to practice, organising youth counselling centres. One such accomplishment was seen when Frankl proudly reported: “That was the first time in many years when no student suicides were reported in Vienna” (Frankl, 2000, p. 68). People in many countries showed interest in his work and invited him to present lectures. During that time, Frankl “gained a treasure chest” of experience (Frankl, 2000, pp. 72–73), developing his professional occupation.

Alexander’s (1988) pointer of Uniqueness can be found regarding Frankl’s opportunity to work as a psychotherapist before graduation:

I tried to forget what I had learned from psychoanalysis and individual psychology so that I could learn from listening to my patients. I wanted to find out how they managed to improve their conditions. I began to improvise (Frankl, 2000, p. 73).

The Destroyer archetype enabled Frankl to let go of anything that no longer supported his growth and find out his true values and meaning. The experience of chaos and uncontrolled power of others over his life led him to explore the lives and perspectives of others and build his more in-depth and reconstructed worldview.

Another example of the Destroyer archetype can be seen after the liberation from a concentration camp when Frankl was informed about his family’s deaths. Frankl’s hope was destroyed, and his survival became meaningless. He wrote that he had “nothing to look for” (Frankl, 2015, p. 51): “Who has no analogous fate, cannot understand me: tired, unspeakably sad, unspeakably lonely, I have nothing to hope for and nothing to fear anymore, I have no joy in life anymore, only duties, I live out my conscience …” (Frankl, 2015, p. 48). He had lost his initial motivation to live and to overcome suffering and experienced powerlessness, disorientation, and meaninglessness. Only years later, he managed to accept mortality and loss and focused on his obligations as a doctor and author: “Nothing has remained—except the responsibility for the spiritual work that I have to fulfil—still and despite, or: perhaps also because I have to suffer so much” (Frankl, 2015, p. 13). Finally, the lost hopes caused by the Destroyer archetype enabled Frankl to re-define his life’s purpose and meaning (Krasovska & Mayer, 2021).

### Archetypal Theory in Frankl’s life: The Caregiver

The archetype of the Caregiver is particularly noticeable in Frankl’s life regarding the relationship with his parents. No recollections are found in his books about the conflicts between his own needs and that of others. The Caregiver occurs in the story regarding an immigration visa: Frankl decided to stay with his parents in Vienna instead of taking his opportunity to go to the USA (Frankl, 2000). This decision put his own life in danger as a Jew; however, he felt obliged in terms of a metaphysical explanation and his conscience to stay with his parents to take care of them (Frankl, 2000).

Another example of the Caregiver occurs during Frankl’s stay in a concentration camp, where he visited his ill father despite the curfew. The morphine he smuggled into the camp was supposed to relieve his father’s pain. After his father's death, Frankl wrote: “[…] I had the most wonderful feeling one can imagine. I had done what I could. I had stayed in Vienna because of my parents, and now I had accompanied Father to the threshold and had spared him the unnecessary agony of death” (Frankl, 2000, p. 26).

The Caregiver archetype, who aims at caring for others beyond one’s own family and friends, is noticeable in Frankl’s professional occupation. Before the deportation and concentration camps, Frankl made efforts to save people from suicide. Frankl recalls his motivation as follows: “I do respect the decision of people to end their lives. But I also wish others respect the principle that I have to save lives as long as I am able” (Frankl, 2000, p. 79). Finally, the archetype of the Caregiver enabled Frankl to find meaning through caring for others despite any danger. In his book “Recollections. An Autobiography” (Frankl, 2000), he mentions the words of John Ruskin, reflecting his attitude towards the suffering of others: “There is only one power: the power to save someone. And there is only one honour: the honour to help someone” (Frankl, 2000, p. 55).

### Archetypal Theory in Frankl’s Life: The Magician

The Magician seems to be the most pronounced archetype throughout Frankl’s life. Already in childhood, Frankl frequently experienced synchronistic life events, which he noticed but was not able to rationalise: “[…] I am too ignorant to explain them and too smart to deny them” (Frankl, 2000, p. 59). An example of such an event is the “hint from heaven” (Frankl, 2000, p. 83). Frankl’s father cited a relevant passage from the Bible at the time when Frankl had to decide on his immigration to the USA (Frankl, 2000). The following examples of decisions resulted from Frankl’s belief in the unconditional meaning of life found throughout his life path (see Krasovska & Mayer, 2021):

1. The will to survive in concentration camps despite any circumstances;
2. Readiness to accept reality, sacrifice the most important, and be humble (the loss of his manuscript in a concentration camp, later the death of his first wife);
3. Doing good deeds for others and not to “vegetate.”

The Magician enabled Frankl to change the physical reality by seeing “beyond the misery of the situation” and “thus to turn an apparently meaningless suffering into a genuine human achievement” (Frankl, 2000, p. 53). Unconditional meaning orientation enabled Frankl not only to stay resilient during suffering, but influence other people not to give up by helping them to discover meaning in suffering.

### Terror Management in Frankl’s Life

Already at a very early age, Frankl dealt with the question of death and whether life had meaning despite human mortality. With this, he addressed a core question of the TMT theory (Barrett, 1958). Frankl came to the understanding that the person should take the responsibility to answer the question of life’s meaning on their own, instead of looking for the answer outside (Frankl, 2000). Early in childhood, Frankl also developed the idea of an ultimate meaning, or suprameaning, which is basically incomprehensible. Since this suprameaning is extremely complex and derives from a higher source, it can only be grasped through faith (Frankl, 2000). This perspective suggests that a human’s life serves a higher goal; life is valuable and has meaning despite suffering and future death. This notion has a crucial meaning for prevention of death-related cognitions when Viktor E. Frankl’s general goals and motivations to survival were challenged. According to Pyszczynski et al. (2010), Frankl dealt with these questions consciously, by relating to the works of Yalom (1980), Greenberg et al. (2004), and Koole et al. (2006).

Throughout his whole life, Frankl lived according to his ideas of life’s meaning as well as suprameaning. His occupation as a physician, psychotherapist, and author helped him to create a meaningful life through serving and doing good deeds for others, despite the feeling of alienation and personal tragedies—to protect himself from the death threat, he adopted a worldview, which protected his worthiness, his self-esteem, and its sustainability, as described by Van Kessel et al. (2020); this created meaning to overcome his existential fears (see Vail et al., 2012). Frankl (2000) was conscious about death as a part of life and showed this, for example, when he decided consciously to stay with his parents in Vienna and risked deportation. He, however, constructed his immortality (see Becker, 1967) through his writing and lectures and through becoming famous.

A strong emotional connection to his family enabled Frankl to preserve his will to live. He felt responsible for his parents and tried to minimise their suffering during World War II (WWII). His love for his first wife, Tilly, strengthened his hope for a better future during the suffering in concentration camps. Finally, when all hopes for a better future were destroyed, the idea of suprameaning helped Frankl to preserve his will to live and cope with existential terror. Tilly and his hopes might be interpreted in a way that they were distractions from death anxiety to create the illusion of immortality through love and a future together with his wife, subsequently, transcending death (Pyszczynski et al., 2010). However, after he had lost his entire family, he faced death and could not minimise fear and effect anymore (Juhl, 2019).

Frankl changed his attitude towards his unchangeable fate by transforming his thoughts to conducting his duties as a doctor and a book author. His transcendent views on life, as well as religious practices (e.g., reading psalms and praying to God), helped him to overcome death anxiety. Death became part of his thoughts more consciously (Hayes et al., 2010) and might have buffered against death anxiety at the same time. As described by Schimel et al. (2007), Frankl increased his prayers and faith to transform his awareness and painful experiences of death. Further, it might be assumed that Frankl’s ability to minimise the negative effects of death anxiety resulted from his childhood experiences with his parents: primarily his father provided him with safety and security, while his mother represented emotional warmth (Frankl, 2000). Secure relational attachment, belief in the meaning in life, might have provided Frankl with a sense of self-worth and buffered his death anxiety even in times of adversity (as in Heine et al., 2006; Vail et al., 2012). In his autobiography, Frankl (2015) writes: “I increasingly realise that life is so infinitely meaningful, that even in suffering and even in failure there must still be meaning” (p. 49). This statement shows that Frankl developed a sense of life’s internal meaning by accepting its shadow and light side and integrating it towards a meaningful whole (Vail et al., 2012).

## Conclusion and Recommendations

This article aimed to explore the meaning of life and death across the lifespan of Viktor E. Frankl utilising two theoretical approaches, namely archetypal theory and TMT. The archetypes and their impact on his life changed across the life span. However, they were impactful symbols creating meaningful throughout his entire life. His views on the meaning of life and death changed over his life and are defined in relation to the archetypes and TMT. He consciously addressed his losses in life, his death anxieties, the pain and the suffering and finally concluded that pain as well as happiness, have an internal and intrinsic meaning that makes life and death meaningful.

Future research should focus on the life of Frankl from different psychobiographical, methodological, and theoretical perspectives to gain a deeper and more holistic view of the complexities of his life. It would be further beneficial to explore the meaning of life in extraordinary individuals, and compare these individuals’ experiences based on comparative psychobiographical approaches.

In applied psychological contexts, psychobiographical works of extraordinary individuals can be used as examples as role models. These works can also be used for client’s increased and in-depth understanding of overcoming suffering and pain and integrating light and shadow experiences across the lifespan. Psychobiographies can finally serve as practical illustrations of coping resources of extraordinary individuals to provide clients in psychotherapy and counselling with new ideas and inspirations.
